# Benchmarking the Builders: A Comparative Analysis of PRosettaC and AlphaFold3 for Predicting PROTAC Ternary Complexes

**DOI:** 10.21203/rs.3.rs-6866610/v1

**Published:** 2025-07-02

**Authors:** Joseph M. Schulz, Sarah I. Schürer, Robert C. Reynolds, Stephan C. Schürer

**Affiliations:** University of Miami; Santa Catalina School; University of Alabama at Birmingham; University of Miami

**Keywords:** PROTACs, AlphaFold, PRosettaC, Computational Modeling, Structure-Based-Drug-Design, Protein Modeling

## Abstract

Targeted protein degradation via PROTACs offers a promising therapeutic strategy, yet accurate modeling of ternary complexes remains a critical challenge in degrader design. In this study, we systematically benchmark two leading structure prediction tools, AlphaFold3 and PRosettaC, against a curated dataset of 36 crystallographically resolved ternary complexes. Using DockQ as a quantitative interface scoring metric, we assess the structural fidelity of predicted complexes under both scaffold-inclusive and stripped configurations.

Our results demonstrate that AlphaFold3’s performance is often inflated by accessory proteins such as Elongin B/C or DDB1, which contribute to overall interface area but not degrader-specific binding. PRosettaC, on the other hand, leverages chemically defined anchor points to yield more geometrically accurate models in select systems, though it frequently fails when linker sampling is insufficient or misaligned. To overcome the limitations of static benchmarking, we introduce a dynamic evaluation strategy using molecular dynamics simulations of the crystal structures. This frame-resolved analysis reveals that several PRosettaC models, while poorly aligned to the static crystal conformation, transiently achieve high DockQ alignment with specific frames along the MD trajectory.

These findings underscore the importance of incorporating protein flexibility into benchmarking workflows and suggest that transient conformational compatibility may be overlooked in conventional evaluations. By combining constraint-based modeling with dynamic frame matching, this study provides a more nuanced framework for assessing ternary complex predictions and informs the selection of *in silico* tools for rational PROTAC development.

## INTRODUCTION

The field of targeted protein degradation (TPD) represents a burgeoning frontier in therapeutic drug design, offering innovative strategies for the selective elimination of disease-related proteins.^1^ Central to this approach is the ubiquitin-proteasome system (UPS), an endogenous cellular pathway that facilitates the breakdown of proteins through a process that tags them with ubiquitin for subsequent proteolysis.^2^ The specificity and precision of the UPS have been leveraged in the development of Proteolysis Targeting Chimeras (PROTACs), a class of bifunctional molecules that recruit an E3 ubiquitin ligase to a target protein, leading to its degradation.^3^ PROTAC technology has thus opened new avenues for addressing challenging targets in drug discovery.^4^

Optimal design of effective PROTAC molecules is contingent upon a deep understanding of the ternary complexes they form with their target proteins and E3 ligases.^5^ Such an understanding has historically been derived from experimental structural biology techniques;^6^ however, the advent of sophisticated *in silico* methods has revolutionized this process.^7–12^ Computational modeling now plays a pivotal role in the initial stages of PROTAC design by predicting the formation and stability of these complexes.

In the current study, we employed two state-of-the-art computational techniques, AlphaFold3 (AF3) and PRosettaC, to model the arrangement of ternary complexes of PROTACs *in silico*.^9,11,13^ Our objective was to compare the capabilities of these tools in predicting the structures of ternary complexes and to validate their accuracy against 36 known crystal structures. To this end, we have utilized DOCKQ v2 (hereafter simply referred to as DockQ), a validated scoring method, to quantitatively assess the predicted interfaces and overall structural congruence with experimentally determined data.^14,15^

Given AF3’s enhanced computational capabilities compared to its predecessor, AlphaFold-Multimer, we set out to explore whether the inclusion of additional scaffold proteins would influence model quality, as assessed by DockQ scores. Specifically, we investigated ternary PROTAC complexes targeting VHL or Cereblon by comparing predictions made with and without accessory proteins – Elongin B and Elongin C for VHL-based systems, and DDB1 for Cereblon-based systems. These scaffold components are known to support proper E3 ligase architecture and may improve structural fidelity in predicted complexes. However, due to residue count limitations on the AF3 server, we were unable to include larger scaffold elements such as cullin-ring ligases or RBX domains, which might have further stabilized the folded conformation during prediction.^16,17^

Our comparative analysis highlights PRosettaC’s improved performance over AF3 in predicting ternary complex geometries that more closely resemble experimentally resolved structures. While the observed differences are modest in magnitude, they are consistent across multiple systems and suggest that PRosettaC may currently offer more reliable structural predictions for PROTAC-focused modeling. These findings are intended to inform the selection of *in silico* tools during the early stages of PROTAC development, particularly for applications where accurate ternary complex modeling can impact downstream design decisions. By contributing a systematic benchmarking of two widely used structural prediction platforms, we aim to support the broader community in refining computational strategies for targeted protein degradation.

## METHODS

### PDB Query and Crystal Structure Selection

For the identification of relevant ternary complexes, a systematic protocol was implemented to query the Protein Data Bank (PDB). Our search parameters were tailored to select structures with a minimum molecular weight criterion of 450 Da for chemical components, indicative of PROTAC presence, and to ensure experimental validation through high-resolution X-ray diffraction. The query was designed to capture a comprehensive set of ternary complex structures, which resulted in the retrieval of 36 unique crystal structures from the literature. These structures served as the empirical benchmarks for our computational predictions and were pivotal for subsequent comparative analyses.

### AlphaFold3 Ternary and Complete Complex Prediction Protocol

Ternary complex models were generated using the latest AF3 server, which supports multimeric protein assembly predictions with high structural fidelity. For each of the 36 benchmarked systems, we submitted two variations: (1) a **Ternary** model consisting solely of the target protein, E3 ligase, and PROTAC degrader sequence, and (2) a **Complete** model, which additionally included accessory proteins known to stabilize the E3 ligase complex – such as Elongin B/C in VHL systems or DDB1 in CRBN systems.

Each input was prepared by concatenating the relevant amino acid sequences without any template guidance or manual restraints. Five models were generated per complex using default AF3 multimer settings. Prediction runtimes were highly efficient, typically completing within 10–30 minutes per submission.

Due to input size constraints imposed by the AF3 server, we did not include larger scaffold proteins such as cullin ring ligases (CUL2, CUL4A) or RING-box domains (RBX1), as their inclusion exceeded the allowable sequence length limits. Instead, we focused on the minimal functional components most relevant to degrader binding and interface evaluation. Resulting models were subsequently scored using DockQ to quantify alignment accuracy with crystal structure references.

### PRosettaC Modeling Technique

PRosettaC, a Rosetta-based protocol specifically designed for modeling PROTAC-induced ternary complexes, was used as the second computational strategy in our benchmarking. This method enforces geometric constraints derived from known warhead binding modes, enabling structure-guided assembly of ternary complexes involving an E3 ligase, a target protein, and a bifunctional degrader.

Inputs to the pipeline included experimentally resolved or modeled structures of the target protein and E3 ligase, along with their respective bound warhead and ligase recruiter. The PROTAC linker was input as a SMILES string, which PRosettaC uses to generate three-dimensional linker conformations compatible with the binding pocket geometries.

To enhance sampling depth beyond the default implementation, we modified the PRosettaC script to permit the generation of up to **1000 models per system**, as opposed to the original 200-model limit. This allowed for broader conformational exploration and improved the likelihood of capturing native-like ternary poses, particularly in systems with flexible or elongated linkers. All models were evaluated using the standard Rosetta energy function, with particular emphasis on total score and interface energy. Final predictions were further ranked by their DockQ alignment to crystal structures.

### DockQ Scoring Methodology

To assess the structural accuracy of our predicted ternary complexes, we employed **DockQ v2**, the recently released Python-based reimplementation of DockQ. This updated version supports automatic chain mapping, multimeric systems, and provides improved speed and portability – features essential for evaluating both AF3 and PRosettaC predictions in our high-throughput workflow.

DockQ v2 was used to score predicted ternary complexes against experimental crystal structures for all 36 systems. Each model was assessed using the composite DockQ metric, which combines interface RMSD (iRMSD), ligand RMSD (LRMSD), and the fraction of native contacts (Fnat) to generate a normalized score between 0 and 1, where higher values indicate closer structural agreement with the reference interface.

In addition to static crystal structure comparisons, DockQ v2 was used to perform **time-resolved evaluations** by comparing predicted models (both from AF3 and PRosettaC) against molecular dynamics (MD) trajectories of the crystal structures. This allowed us to measure alignment across thousands of frames, revealing whether any models transiently matched conformations observed in solution. For PRosettaC, which can produce up to 1000 models per complex following our script modification, this enabled fine-grained evaluation across diverse structural snapshots.

### Molecular Dynamics Setup and Simulation Protocol

To assess the dynamic behavior and conformational stability of modeled ternary complexes, we performed all-atom MD simulations using GROMACS 2023.1. The CHARMM36-jul2022 force field was employed for all protein and ligand components to ensure compatibility with high-fidelity protein–ligand interactions. Ligand parameters were generated using the CGenFF server, with .str files converted to GROMACS-compatible .itp formats.

#### A System Preparation and Solvation

Each complex – whether derived from experimental crystal structures or computational models – was first converted into GROMACS format and assigned appropriate protonation states. Systems were solvated in a TIP3P water box with a 1.0 nm buffer and neutralized with Na+ and Cl^−^ ions to achieve an ionic strength of 0.15 M. Topologies were generated using standard CHARMM36 force field protocols.

#### B Energy Minimization and Equilibration

After solvation, systems underwent steepest descent energy minimization until a maximum force threshold of 1000 kJ/mol/nm was reached. Equilibration was conducted in two phases: a 100 ps NVT (constant Number, Volume, Temperature) ensemble using the modified Berendsen thermostat at 300°K, followed by a 100 ps NPT (constant Number, Pressure, Temperature) ensemble using the Parrinello–Rahman barostat at 1 bar. Position restraints were applied to heavy atoms of the protein and ligand during equilibration to allow solvent relaxation.

#### C Production Simulations

Production runs were performed in the NPT ensemble for **50 ns** per system using a 2 fs integration time step and LINCS constraints on all bonds. Long-range electrostatics were treated using the Particle Mesh Ewald (PME) method. Periodic boundary conditions were applied in all directions, and coordinates were saved every 10 ps, yielding 5000 frames per trajectory for downstream analysis.

All simulations were conducted on GPU-accelerated high-performance computing clusters to ensure efficient sampling. Trajectories were post-processed to correct for periodicity and protein displacement, allowing accurate computation of root-mean-square deviation (RMSD), root-mean-square fluctuation (RMSF), and frame-by-frame DockQ comparisons to predicted and experimental structures.

### Statistical Analysis

To comprehensively assess the structural accuracy of predicted ternary complexes, we conducted both descriptive and inferential statistical analyses across multiple modeling strategies: AF3 Ternary, AF3 Complete, AF3 Complete* (scaffold-stripped), and PRosettaC. DockQ v2 scores were analyzed at the per-model and per-structure level to evaluate interface quality across 36 crystal benchmark systems.

Descriptive statistics – including median, mean, standard deviation, and interquartile range – were calculated for each method to summarize performance distributions. To assess significance in model performance, **paired Wilcoxon signed-rank** tests were applied to compare median DockQ values across methods (e.g., PRosettaC vs AF3 Ternary, Complete vs Ternary), with **Cohen’s d** used to estimate effect sizes.

Visualization of score distributions was achieved through **box plots, swarm plots, scatter plots**, and **heatmaps** of per-complex deltas, allowing intuitive comparison across prediction strategies. Additionally, **ranking frequency plots** and **median-centered bar charts** were used to compare relative method performance across systems.

For MD simulations, **time-series DockQ trajectories** were generated across 5000 MD frames for five benchmark systems. We analyzed per-frame fluctuations in DockQ scores for each AF3 Ternary model (models 0–4), annotating peak scores and comparing temporal trends to assess whether any spontaneous realignment toward the crystal interface occurred. Maximum observed DockQ scores were noted for each trajectory, and graphical overlays were used to highlight these transient structural alignments.

In a follow-up dynamic evaluation, we also compared **static PRosettaC models against MD-resolved frames** of the crystal structures to detect transient conformational matches, enabling a frame-by-frame similarity analysis that captured model–receptor alignment beyond the static crystal pose.

All statistical analyses were performed using Python libraries including scipy.stats, pandas, and matplotlib, and visualizations were rendered using seaborn and plotly.

## RESULTS

### I. Comparative Performance of AlphaFold3 Ternary and Complete Complex Predictions

To assess how system composition influences the predictive fidelity of AF3, we benchmarked two modeling strategies across a curated dataset of 36 crystallographically resolved ternary complexes. The Ternary Complex models were constructed using only the core components – target protein, E3 ligase, and PROTAC molecule – whereas the Complete Complex models incorporated additional co-resolved accessory proteins, such as elongin B/C (VHL-based systems) and DDB1 (CRBN-based systems).

#### Overall Performance Trends.

The Complete Complex models consistently outperformed the Ternary counterparts, both in median and model-level DockQ scores. As shown in **Figure S1**, Complete predictions often achieved DockQ values exceeding 0.8 – classified as “high-quality” in the CAPRI criteria – while Ternary models rarely surpassed 0.2. This trend was preserved across nearly all benchmark systems. The improvement was particularly striking for complexes such as 7KHH, 5T35, and 8BEB, where median scores rose from < 0.1 in Ternary models to > 0.9 in the Complete condition.

#### Per-Complex Comparison.

The comparative advantage of Complete modeling is further illustrated in **Figure S2**, which traces the median DockQ score per structure for both model types. While the Ternary predictions hovered at the low end of the spectrum for the majority of cases, the Complete models exhibited a dramatic upward shift, often approaching perfect alignment with native interfaces. The steep vertical deltas between paired lines highlight the magnitude of improvement upon inclusion of cofactors. Importantly, **32 out of 36** structures exhibited a positive median delta in favor of the Complete configuration, suggesting this is not a dataset-specific artifact but rather a general feature of PROTAC ternary complex prediction.

#### Model-Level Distribution and Reproducibility.

To probe intra-complex variability, we plotted all individual DockQ scores in a swarm plot (**Figure S3**). Complete models displayed tight, consistent clusters near the high end of the DockQ scale, indicating both **accuracy and reproducibility**. In contrast, Ternary models showed considerable variance, often failing to converge on a meaningful interface. This suggests that the presence of structural scaffolds (e.g., elongin subunits) helps stabilize AF3 predictions by preserving spatial context.

#### Quantitative Differences and Distributional Shifts.

The magnitude of improvement was visualized as a heatmap of per-complex median deltas (Complete – Ternary) in **Figure S4**. The majority of complexes are shaded deep red, indicating robust DockQ gains. Only a handful of complexes showed negligible or slightly negative differences, and in those cases, both model types performed poorly overall, likely reflecting intrinsically challenging systems. Complementing this, **Figure S5** plots individual scores as a scatter distribution, clearly demonstrating the bifurcation in performance. Complete models populate the upper right quadrant of the plot with high-scoring consistency, while Ternary predictions appear scattered and predominantly low.

#### Statistical Validation.

A paired t-test comparing the median DockQ scores per structure yielded a statistically significant difference between the two modeling strategies (T = − 5.55, p = 3.02 × 10^−6^). This affirms that the observed performance gains are not due to sampling variance but rather reflect a reproducible structural advantage imparted by full-complex input modeling.

### II. Assessing the Contribution of Accessory Interfaces to DockQ Gains

While the superior performance of Complete models in reproducing crystallographic interfaces was evident (Figures S1–S5), the magnitude of this improvement raised the possibility that the observed gains may not reflect improvements in E3-target interactions *per se*. Instead, it was hypothesized that the additional proteins retained in Complete models – most notably Elongin B/C in VHL-based complexes and DDB1 in CRBN systems – might introduce spurious interface contacts that artificially inflate DockQ scores. To interrogate this, we curated a subset of the Complete dataset by removing all accessory proteins prior to DockQ evaluation, generating a new control group termed Complete* (i.e., stripped models evaluated against the native interface only).

Across the dataset, cleaning out accessory elements dramatically reduced DockQ scores, often collapsing previously high-scoring models into the low-accuracy regime (**Figure S6**). Boxplots comparing per-complex DockQ distributions showed substantial downward shifts in Complete* models across nearly all systems, with prominent drops observed in structures such as 7KHH, 6BOY, 6BN7, and 8QVU, which had originally ranked among the top performers in the Complete group.

To quantify the effect of structure cleaning, a cumulative distribution function (CDF) of DockQ scores was plotted for both Complete and Complete* datasets (Figure S7). This revealed a leftward shift in the entire distribution upon accessory removal, with over 90% of Complete* models scoring below 0.2, in stark contrast to the multimodal distribution of the original Complete models. Additionally, the majority of Complete* models cluster in the lowest scoring bin (0.0–0.1), indicating failure to recapitulate native-like interfaces after stripping accessory proteins (**Figure S8**). In contrast, the uncleaned Complete models show a broader distribution, with a significant fraction achieving DockQ scores > 0.8, suggesting that inclusion of accessory components improves predicted interface quality in many cases.

Further, a bar plot of DockQ delta values (Complete* – Complete) highlighted the per-model impact of cleaning (**Figure S9**). Nearly all models showed negative shifts, with many complexes exhibiting mean deltas exceeding − 0.9. When median DockQ values were analyzed per complex (**Figure S10**), the same pattern emerged: 25 out of 26 complexes displayed a reduction in central tendency, with the largest losses occurring in those known to involve multicomponent ligase scaffolds. This strongly suggests that prior improvements in DockQ were in many cases confounded by off-target interactions involving non-target subunits.

Notably, a side-by-side scatter plot of DockQ scores from both conditions (**Figure S11**) visualized this collapse at the individual model level. Cleaned structures (green) consistently underperformed their unstripped counterparts (orange points), with minimal overlap in the high-DockQ range. These results provide strong evidence that accessory subunits – despite being biologically relevant – introduce confounding surfaces that distort the evaluation of ternary complex fidelity.

Statistical analyses confirmed this observed shift. A paired t-test yielded a T-statistic of − 12.88 (p = 2.78×10^−27^), and a nonparametric Wilcoxon signed-rank test yielded p = 5.58×10^−20^, reinforcing that the DockQ decline following structure cleaning is a significant and systematic outcome of interface disruption, rather than stochastic variation.

### III. Comparative Performance of PRosettaC and AlphaFold3 Variants

To extend our benchmarking beyond AF3-native modeling strategies, we evaluated PROTAC complex predictions generated using PRosettaC – a constraint-guided Rosetta-based protocol for ternary assembly. Due to limitations associated with ligand or chain mapping in the original PRosettaC script, we were unable to model 11 of the 36 systems. The reasons for these failures (e.g., unresolvable chain conflicts, linker atom mismatches, or fragment docking crashes) are summarized in the Discussion section. Here, we restrict our analysis to the 25 systems that were successfully modeled using all three methods: PRosettaC, AF3 Ternary, and AF3 Complete*.

**Figure 1:**
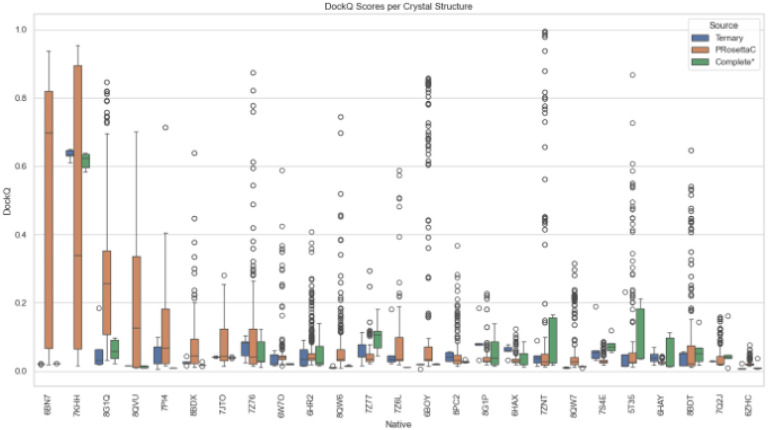
Median-centered DockQ score distributions per method for each crystal structure. PRosettaC displays broader score variability and higher medians in several cases, especially 6BN7, 6ZHC, 7KHH, and 8QVU. AF3 Complete* and Ternary predictions cluster tightly at low DockQ values.

Across these 25 structures, we compared DockQ scores to quantify the accuracy of predicted interfaces relative to experimental crystal structures. PRosettaC predictions yielded a notably broader distribution of DockQ values than either AlphaFold variant (Fig. 1). In several cases – most prominently 6BN7, 6ZHC, 7KHH, and 8QVU – PRosettaC achieved high-scoring outliers or consistently superior medians. AF3 Complete* tended to yield tighter, low-variance distributions concentrated in the 0.02–0.12 range, while Ternary predictions were more uniform but typically low-scoring. These findings suggest that PRosettaC’s constrained sampling mechanism is capable of accessing productive poses not readily generated by end-to-end neural models, particularly in well-defined anchor geometries.

**Figure 2:**
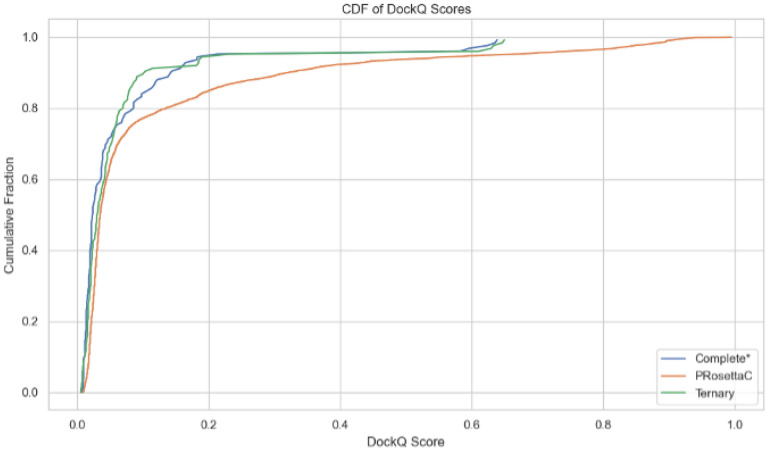
Cumulative distribution function (CDF) of DockQ scores across all predictions. AF3 Ternary and Complete* models exhibit highly skewed distributions toward low DockQ values, while PRosettaC maintains a longer high-score tail.

**Figure 3:**
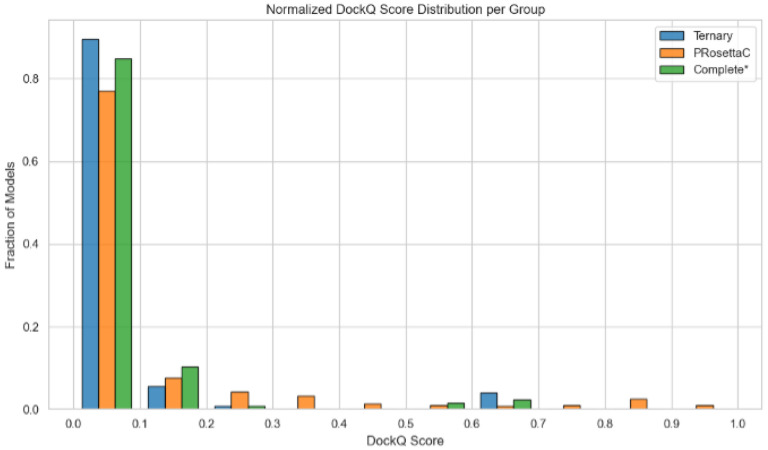
*Normalized distribution of DockQ scores across three prediction methods: Ternary (AlphaFold-based minimal complex), PRosettaC (Rosetta-based docking), and Complete*(AlphaFold complex stripped of scaffold proteins). Each bar represents the fraction of models within a given DockQ bin, normalized within each method group. The majority of models across all methods fall below the 0.1 DockQ threshold, though PRosettaC and Complete* show a broader distribution and slightly higher frequencies in mid-to-high score bins, reflecting improved modeling of native-like interfaces in certain systems.*

To visualize DockQ score coverage more holistically, we constructed cumulative distribution functions (CDFs) for each method (Fig. 2). The AF3 Ternary and Complete* models concentrated most predictions within the lower DockQ regime (< 0.2), while PRosettaC preserved a longer high-scoring tail. Notably, PRosettaC surpassed the 0.23 “acceptable” DockQ threshold more frequently than AF3 variants (Fig. 3). These data reinforce the notion that constraint-guided docking retains the ability to generate native-like interactions even when the median performance is modest.

**Figure 4:**
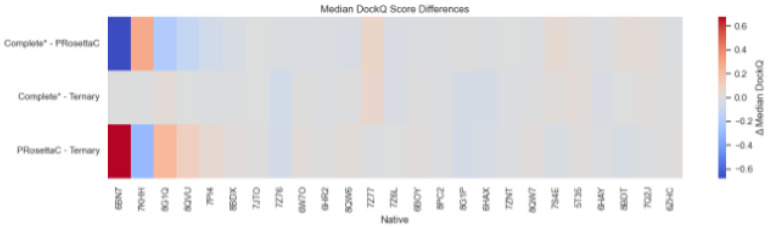
Median DockQ score differences between prediction methods for each benchmark target. *Each row indicates a pairwise comparison between methods (e.g., PRosettaC vs Ternary), and each column corresponds to a native crystal structure (PDB ID). Cell colors represent ΔDockQ, calculated as the difference between the row method and the other method in the comparison. Warmer colors (red) indicate that the row method outperformed the comparator; cooler colors (blue) indicate the opposite. PRosettaC consistently outperforms AF3 Ternary, while AF3 Complete* models occasionally show superior performance on scaffold-stabilized systems.

We next assessed the relative performance of each method through pairwise comparisons of median DockQ scores across benchmark structures. As shown in the delta heatmap (Fig. 4), PRosettaC consistently outperforms AF3 Ternary models, with an average ΔDockQ of + 0.11, indicating a marked improvement in interface prediction when using Rosetta-based docking. In contrast, AF3 Complete* models sometimes outperform PRosettaC, particularly for targets such as 7PI4 and 8G1Q, suggesting that AF3 benefits from multimeric context or scaffold-stabilized configurations in specific systems. These results highlight PRosettaC’s strengths in sampling realistic ternary poses, while also illustrating the contexts in which AF3 predictions – when scaffolded – can be more effective.

To better visualize model-level variability, we plotted all individual DockQ scores per native complex using dot plots and swarm plots (**Figures S12** and 5). PRosettaC generated a wide spectrum of poses across most systems, with several high-scoring outliers that far exceeded AF3 predictions. These standout examples suggest that while PRosettaC can struggle in difficult scenarios, it maintains potential for highly accurate predictions in favorable contexts.

**Figure 6:**
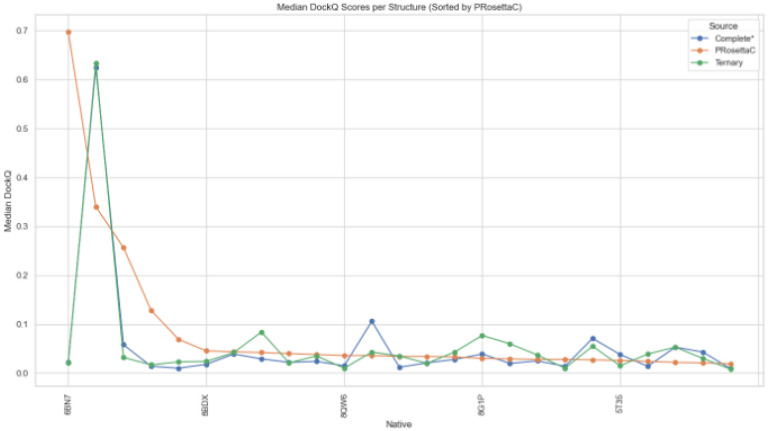
Median DockQ score per method for each native complex. PRosettaC outperforms in nearly half of the targets, with some competition from Complete* (e.g., 5T35) and Ternary (e.g., 6HAX).

We then calculated the median DockQ per method and visualized these values by target in a grouped line plot (Fig. 6). On a per-target basis, the method with the highest median was considered the “winner” and assigned a rank. These rankings were summarized in a heatmap (**Figure S13**), highlighting the spread of first-, second-, and third-place finishes.

To summarize overall method performance, we generated a stacked bar chart of ranking frequencies (**Figure S14**). PRosettaC was the top-performing method in 48% of the cases, followed by AF3 Ternary (32%) and Complete* (20%). These results highlight PRosettaC’s dominant, though not universal, advantage in ternary complex prediction. =

### IV. Stability of AlphaFold3 Ternary Predictions Under MD Simulation

To further interrogate the predictive stability of AF3 Ternary models, we performed 50-nanosecond MD simulations on five crystallographically resolved PROTAC complexes. The purpose of this analysis was to evaluate whether relaxation through MD would enhance the alignment of the AF3-predicted interface with the native crystallographic pose, as quantified by DockQ score over time.

Overall, DockQ values remained consistently low throughout all trajectories, indicating minimal spontaneous convergence toward the experimentally resolved geometry. In each system, we tracked DockQ scores per frame for each AF model (models 0–4) to assess structural drift or alignment improvement post-relaxation.

**Figure 7:**
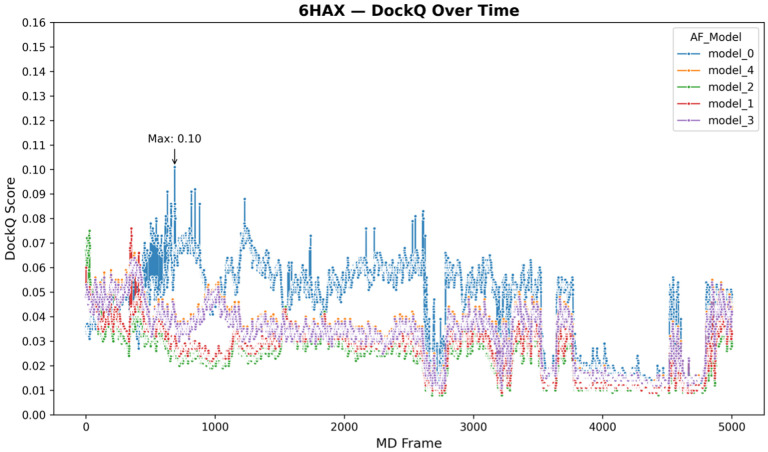
Time-resolved DockQ scores for the 6HAX (SMARCA2:VHL) crystal complex across all AF3 Ternary models during 50 ns MD simulations. Each colored line corresponds to one of the five AF3 models, tracking the DockQ score across 5000 frames. The highest observed DockQ score, 0.10, is reached early in the trajectory by model_0 near frame 1000, marked by a sharp but isolated spike. Despite these transient elevations, no model exhibits sustained improvement or convergence toward a crystal-like interface. Overall, the system remains highly dynamic and fluctuating, with all models oscillating below the 0.1 accuracy threshold across the simulation.

6HAX and 6HAY, both targeting the SMARCA2 bromodomain with a VHL E3 ligase showed similar temporal profiles. Both exhibited significant early fluctuation across AF models, including brief local DockQ increases (approaching 0.05–0.08) around frames 900–1100. However, no sustained improvement was observed, and all models remained below the DockQ threshold of 0.1 throughout the simulation (Figs. 7 and S15).

6HR2, a SMARCA4-targeting VHL complex, displayed the most stable DockQ progression across all models. While small increases were visible in the latter half of the trajectory (particularly for models 1 and 3), DockQ values never exceeded 0.07, remaining well within the low-confidence regime (**Figure S16**).

In the BTK-targeting system 8DSO, a modest upward trend was observed for select models during the mid-simulation window (frames 1500–2500), with transient peaks in the 0.06–0.07 range. However, scores subsequently declined or plateaued, indicating a lack of consistent structural improvement (**Figure S17**).

Finally, 8QVU, a KRAS-targeting complex, demonstrated the least variation across the board. DockQ values for all models were tightly clustered between 0.015–0.045, showing no meaningful improvement or deviation throughout the simulation **(Figure S18**). This suggests a highly stable but poorly aligned conformation.

Collectively, these results indicate that even with extended MD sampling, AF3 Ternary models fail to improve their structural agreement with crystal structures. This reinforces the notion that without explicit structural constraints or multimeric context, AF3 predictions for ternary complexes do not readily refine themselves toward biologically relevant poses – even when allowed full atomistic flexibility.

### V. MD-Resolved Evaluation of PRosettaC Predictions Against a Flexible Crystal Reference Reveals Transient High-Fidelity Conformations

Previous assessments of PRosettaC model accuracy relied on comparisons to a single static crystal structure, typically as deposited in the RCSB Protein Data Bank (PDB). However, these static structures capture only one conformation from what is inherently a dynamic ensemble of states accessible to proteins in solution. This rigid representation may fail to account for transient geometries that are compatible with plausible degrader-mediated ternary complex formation.

To better evaluate the conformational compatibility of PRosettaC predictions, we subjected the crystal structures of four benchmark complexes (6HAX, 6HAY, 6HR2, and 8QVU) to MD simulations. We then compared each PRosettaC model not to a single structure, but to a time series of receptor conformations extracted from the MD trajectory, sampled every 50 frames. This enabled a **frame-resolved similarity analysis** using DockQ as a structural comparison metric between the predicted ternary complexes and the dynamic crystal complex at each sampled frame.

This dynamic comparison approach uncovered a layer of structural nuance not visible in the original static evaluation. While most PRosettaC predictions scored poorly against the crystal structure alone, several showed significantly improved DockQ alignment with specific frames of the MD simulation. These transient peaks in DockQ score suggest that some PRosettaC predictions are more compatible with rare or short-lived conformations of the receptor, underscoring the limitations of relying solely on static reference structures for degrader design assessment.

For example, in the KRAS:VHL system (8QVU), DockQ scores as high as 0.69 were observed when comparing PRosettaC predictions against certain MD frames – substantially higher than scores observed when using the static crystal reference (**Figure S19**). Similarly, the SMARCA4:VHL complex (6HR2) showed a broader range of moderate-to-high scores, with multiple predictions exceeding 0.5 at different time points (**Figure S20**). These data suggest that PRosettaC may be capturing valid ternary geometries that are only transiently compatible with the receptor’s conformational landscape. In contrast, systems like 6HAY (SMARCA2:VHL) maintained low scores throughout most of the trajectory (**Figure S21**), indicating limited model–structure agreement even in flexible contexts. 6HAX, another SMARCA2:VHL system, revealed scattered high-scoring outliers (DockQ > 0.4) despite a majority of low-scoring frames, suggesting episodic compatibility in rare conformations (**Figure S22**).

**Figure 8:**
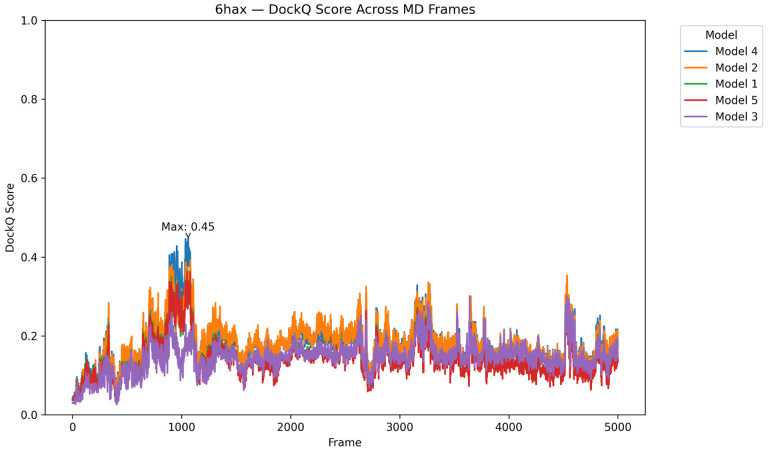
DockQ scores of the top 5 PRosettaC models for the SMARCA2:VHL ternary complex (PDB 6HAX) measured across MD simulation frames of the crystal complex. Each line represents a different PRosettaC prediction, evaluated against the dynamic trajectory of the receptor complex sampled every 50 frames. While most frames yield low DockQ scores, multiple high-scoring episodes (DockQ > 0.4) emerge sporadically, indicating transient structural compatibility between modeled ternary complexes and rare receptor conformations observed during the simulation.

To complement our system-wide evaluation, we further investigated the time-resolved performance of the top five PRosettaC models for the 6HAX system (Fig. 8). By tracking DockQ scores across the full MD trajectory for each individual model, we observed that while baseline scores remained modest for most frames, several models exhibited distinct high-scoring bursts (DockQ > 0.4) aligned with transient conformational states of the receptor. This reinforces the notion that static evaluations alone may undervalue models that are compatible with dynamic receptor geometries. Notably, some models reached their peak DockQ score at different time points, indicating diverse modes of transient fit rather than convergence on a single optimal conformation. These insights illustrate the utility of frame-wise MD alignment not only as a benchmarking tool, but also as a lens for uncovering hidden compatibilities that could guide degrader optimization.

## DISCUSSION

Designing therapeutics that operate through targeted protein degradation poses unique structural challenges. PROTACs function by co-opting cellular ubiquitination machinery, requiring the formation of a stable ternary complex between a target protein, an E3 ligase, and the degrader molecule.^18^ Modeling this assembly *in silico* is non-trivial, as it depends on both the global stability of the multi-protein architecture and the precise geometry of linker-mediated interfaces.^19^ Our study presents a systematic comparison of two leading computational strategies – AF3 and PRosettaC – across 36 crystallographically validated ternary complexes. By evaluating them across static, scaffold-aware, and dynamic simulation contexts, we uncover fundamental tradeoffs in modeling assumptions, interface accuracy, and evaluation methodology (Table 1).

AF3 and PRosettaC embody fundamentally different design philosophies. AF3 models ternary complexes holistically, optimizing for a global free energy minimum using deep neural network priors. This makes it well-suited to discovering plausible overall folds, even in the absence of prior structural knowledge. However, its lack of degrader-specific constraints often results in protein orientations that preclude physical linker placement between E3 ligase and target, despite achieving high confidence scores and structurally coherent global assemblies.^20^

PRosettaC, in contrast, builds from known degrader warheads. It explicitly enforces anchor atom geometries derived from known binding modes and samples linker conformations that connect these anchors. This approach yields highly relevant interface predictions when anchor chemistry is compatible – but is brittle when the anchor distance, orientation, or sampling strategy fails to produce viable geometries. In such cases, PRosettaC produces either nonphysical complexes or fails entirely to generate a model.

This contrast highlights a broader modeling tradeoff: AF3 provides robustness through flexibility, while PRosettaC offers precision through constraint. Choosing between them depends on the design context – whether the goal is exploratory modeling of unknown degrader scaffolds, or fine-grained optimization of linker orientations in known systems.

Across our benchmark, AF3 consistently produced high DockQ scores in its full “Complete” configuration. However, these scores were often inflated by non-relevant interactions – particularly contacts involving accessory scaffold proteins like Elongin B/C or DDB1, which are not directly involved in the degrader interface. When these scaffolds were removed in the “Complete*” configuration, DockQ scores dropped significantly, revealing that much of AF3’s perceived success was due to tertiary structure context rather than degrader-driven binding geometry.

This underscores a critical limitation of global structural similarity metrics like DockQ: they reward total interface overlap, even if the interacting surfaces are irrelevant to the function of interest. For degrader design, where the functional interface lies specifically between the ligase, linker, and target, global metrics must be interpreted cautiously. High scores do not necessarily imply chemically actionable poses.

PRosettaC’s performance, when successful, was not only consistent with known warhead geometries but also scored competitively under these global metrics. In several cases, it produced models that matched or exceeded the accuracy of AF3, despite using a narrower modeling scope. This indicates that constraint-driven docking can yield superior results when prior knowledge is available and compatible.

PRosettaC’s failure modes are instructive. In 11 of the 36 systems, the pipeline could not generate viable ternary complexes. Most failures stemmed from the linker sampling stage, where anchor atoms could not be connected within the physical tolerances defined by the protocol. This often occurred in systems with unconventional binding topologies – where warheads adopted folded, flipped, or orthogonal orientations that defied linear interpolation. PRosettaC’s reliance on point-to-point distance constraints made it ill-equipped to sample such geometries.

This rigidity reflects a broader challenge in degrader modeling: the spatial path between ligase and target is rarely straight. Flexible linkers often adopt convoluted shapes, especially in cases involving deep binding pockets or allosteric surfaces. Addressing these challenges may require next-generation sampling algorithms – such as torsion-driven, ensemble-based, or MD-informed conformer generation – to more fully explore the solution space.

Standard structural benchmarks compare model predictions to a single crystallographic conformation. But crystal structures represent static snapshots, often averaged across dynamic ensembles. To better assess the plausibility of modeled poses, we introduced an MD-based comparison framework that treats the crystal structure itself as dynamic – extracting thousands of trajectory frames from a 50 ns simulation of each crystallographic ternary complex.

This allowed us to evaluate whether any PRosettaC models, even those with poor static DockQ scores, transiently aligned with native-like conformations during simulation. The results were striking. In multiple systems (e.g., 6HR2, 8QVU), PRosettaC models that scored poorly against the static crystal nevertheless achieved high DockQ similarity (up to 0.69) to certain frames in the dynamic reference. Similarly, for 6HAX, the highest DockQ score against the static crystal was under 0.2, but several PRosettaC predictions reached as high as 0.42 when evaluated against MD-sampled frames, indicating strong episodic alignment with transient native-like geometries.

This insight is significant. It means that structural fidelity should not be judged solely by proximity to a single reference structure, but instead contextualized within the full range of biologically plausible conformations. A model that transiently aligns with an accessible conformation may be as valuable for drug design as one that perfectly matches a crystallographic pose.

Despite the comprehensive scope of this benchmark, several limitations should be acknowledged.

First, the use of the web-based AF3 server imposed residue count restrictions that prevented inclusion of full-length E3 ligase complexes, such as CUL2, CRL4A, and RBX1. While the accessory proteins Elongin B/C and DDB1 were included in the Complete models, the omission of larger scaffold components may reduce structural realism in multimeric systems and limit the generalizability of the results.

Second, the PRosettaC protocol failed to generate viable models in 11 out of the 36 benchmarked systems. These failures were primarily due to linker sampling constraints, unresolved atom naming inconsistencies, or warhead misalignments. These issues reflect the inherent brittleness of constraint-based modeling when faced with non-canonical anchor geometries or complex topologies.

Third, the DockQ scoring metric, while valuable for quantifying structural alignment, can be biased when large scaffold proteins dominate the predicted interface. In such cases, scores may overstate model quality without necessarily capturing degrader-relevant interactions. Although our analysis employed a scaffold-stripped evaluation (Complete*) to mitigate this issue, the metric remains agnostic to functional binding site relevance unless custom interface filtering is applied.

Fourth, the benchmarking framework primarily relied on static crystallographic structures as reference points. However, MD simulations revealed that some models – particularly from PRosettaC – aligned well with specific frames in the dynamic ensemble, despite scoring poorly against the static crystal pose. This suggests that conventional evaluations may underestimate the biological plausibility of certain conformations.

Finally, the study lacks direct experimental validation of model function. While DockQ scores and MD-based frame comparisons offer robust structural insights, no cellular degradation assays or biochemical data were used to confirm the efficacy or relevance of the predicted ternary complexes. Future studies should incorporate orthogonal experimental validation to fully assess the predictive power of these computational tools.

While this study primarily evaluates model fidelity using structural metrics and MD simulations, future work should integrate functional validation. Specifically, degradation assays could be employed to assess systems where PRosettaC-generated models exhibit strong alignment with high-fidelity MD frames despite low static DockQ scores. These cases suggest that dynamic compatibility – not just crystallographic resemblance – may underlie effective ternary complex formation and degrader function. Validating such predictions in cellular or biochemical degradation assays could help refine structural benchmarks and improve the real-world utility of modeling tools in PROTAC design.

Together, our results highlight the need for degrader-specific structural evaluation strategies. AF3 offers speed and flexibility but lacks precision at the degrader interface. PRosettaC delivers interface-relevant models when constraints are satisfied but fails when sampling is inadequate. DockQ, while convenient, can mislead when scaffold proteins dominate the contact surface or when dynamic flexibility is ignored.

We propose that future benchmarking pipelines incorporate:

- Frame-averaged or ensemble-based similarity scoring
- Interface-partitioned metrics that isolate degrader-relevant contacts
- Assessment of linker strain, burial, and solvent exposure
- Torsion-aware sampling methods to explore non-canonical geometries

Ultimately, successful PROTAC modeling must blend structural fidelity with functional awareness. Our findings suggest that hybrid strategies – combining global fold predictors with degrader-specific docking constraints – may offer the best path forward. As experimental validation remains the gold standard, our computational toolkit must evolve to reflect the true complexity of the degradome landscape.

## Supplementary Files

This is a list of supplementary files associated with this preprint. Click to download.

- SupplementalFinal.docx

## Figures and Tables

**Figure 5:**
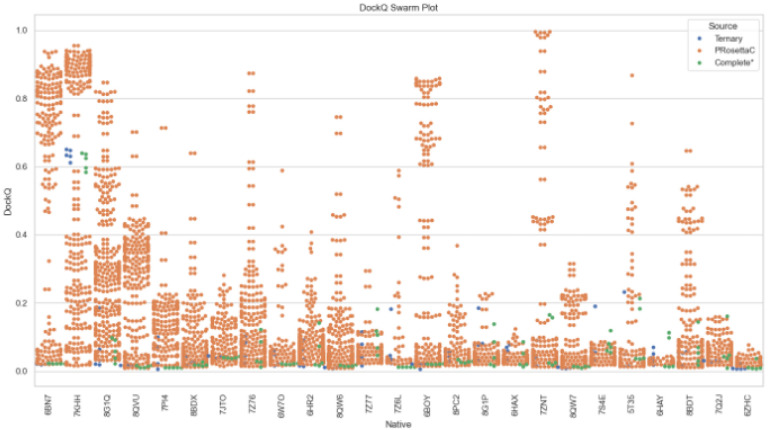
Swarm plot of DockQ scores per model. PRosettaC consistently populates the high-scoring regime (>0.5), particularly for 6BN7, 6ZHC, and 7KHH, while AF3 methods cluster near baseline.

**Table 1:** Comparative Summary of Ternary Complex Modeling Methods.

Criterion	AlphaFold3 Ternary	AlphaFold3 Complete*	PRosettaC
Successful Models (out of 36)**	36	36	25
DockQ > 0.5 (High-Fidelity Models)	5 of 180 models (2.8%)	5 of 180 models (2.8%)	527 of 8,407 models (6%) – concentrated in a few favorable systems
Use of Structural Cofactors	No	Yes	No
Degrader-Aware Modeling	No	No	Yes (warhead-guided)
Scaffold Bias in DockQ	No	Yes	No
Linker Geometry Enforcement	No	No	Yes
Sensitivity to Protein Flexibility	Low	Low	Medium–High
Improves During MD Simulation	No	No	Yes (via frame alignment)
Sampling Failure Rate	0%	0%	~ 31% (11/36 systems failed)

Overview of key performance characteristics across AF3 Ternary, AF3 Complete* (scaffold-stripped), and PRosettaC modeling strategies. Complete* models were derived by removing accessory proteins (e.g., Elongin B/C, DDB1) from AF3 Complete predictions prior to DockQ evaluation, isolating degrader-specific binding interfaces. The table highlights modeling success rate, interface constraints, flexibility handling, and dynamic compatibility. While PRosettaC excels in degrader-specific modeling and transient alignment with MD frames, its sampling failures in certain geometries remain a challenge. AF3 Complete* models expose how much scoring previously relied on scaffold-derived contacts, often revealing limited degrader-target interaction fidelity when scaffolds are stripped.

** DockQ scores for AF3 Complete* reflect stripped scaffold structures. Success is defined as generation of parsable models suitable for scoring.

## Data Availability

The datasets used and/or analyzed during the current study are available from the corresponding author on reasonable request.
